# Impact of immunoadsorption on autoantibodies and physical performance in post-COVID patients: a prospective exploratory study of 18 participants

**DOI:** 10.1007/s15010-026-02914-8

**Published:** 2026-08-11

**Authors:** Christine Wossidlo, Felix Steinbeck, Hilte Geerdes-Fenge, Martina Sombetzki, Steffen Mitzner, Micha Loebermann, Emil C. Reisinger, Sebastian Koball

**Affiliations:** 1 Department of Internal Medicine, Division of Infectious Diseases, Nephrology, Endocrinology and Tropical Medicine, University Medical Center Rostock, Rostock, Germany; 2 Core Facility for Cell Sorting and Cell Analysis, University Medical Center Rostock, Rostock, Germany

**Keywords:** Immunoadsorption, COVID-19, Post-COVID, Autoantibodies, Adrenergic receptors, Muscarinic receptors

## Abstract

**Purpose:**

Autoantibodies against β-adrenergic and muscarinic acetylcholine receptors are said to play a role in the pathophysiology of post-COVID syndrome. To date, only a limited number of studies have investigated the effectiveness of immunoadsorption. This prospective exploratory study investigates the effectiveness of immunoadsorption by assessing reductions in autoantibody levels and improvements in physical performance.

**Methods:**

In a prospective exploratory clinical trial, 18 patients with post-COVID syndrome and elevated levels of antibodies against β1/β2-adrenergic receptors and/or M3/M4-muscarinic acetylcholine receptors were treated with a course of five consecutive immunoadsorption sessions. Clinical assessments, objective functional performance tests (e.g., step counters), laboratory parameters, and self-reported performance were performed during the study.

**Results:**

A total of 15 women and 3 men were included in the study. Immunoadsorption was well tolerated. All participants showed a significant reduction in circulating antibodies against β1/β2-adrenergic receptors and M3/M4-muscarinic acetylcholine receptors; however, these levels returned to baseline within the following 3 to 6 months. Objective determinations of physical performance showed no significant improvement following immunoadsorption. Self-reported evaluation revealed a slight improvement in pain and fatigue. However, the correlation analysis failed to demonstrate a significant association between changes in autoantibody levels and subjectively perceived symptoms.

**Conclusion:**

Immunoadsorption was safe and resulted in a short-term, significant reduction in measured autoantibodies. However, there were no improvements in objectively measured performance. Our results indicate that immunoadsorption is not an effective approach for patients with post-COVID syndrome.

## Introduction

The COVID-19 pandemic continues to have profound impacts on society and global healthcare systems. As of May 10, 2026, the World Health Organization reported 779 million confirmed cases of COVID-19 and more than 7 million deaths [1]. While at the early stages of the pandemic the acute infection and its immediate complications were the primary causes of concern, attention shifted to long-term health consequences as the pandemic progressed.

Post-COVID syndrome, also known as Long COVID or Post-Acute Sequelae of SARS-CoV-2 Infection (PASC), encompasses symptoms that persist or emerge weeks to months after an acute SARS-CoV-2 infection and may significantly impair the quality of life of affected individuals [2]. More than 200 symptoms have been described in association with post-COVID in the literature [3]. The four key symptoms of fatigue, cardiopulmonary complaints such as dyspnea and tachycardia, cognitive impairments, and pain form typical clinical phenotypic clusters around which other complaints are grouped. Some patients also report olfactory disturbances, as well as post-exertional malaise (PEM), which is often presumed to be associated with Myalgic Encephalomyelitis/Chronic Fatigue Syndrome (ME/CFS) [4]. The symptoms may persist for months or years, and each subsequent SARS-CoV-2 infection increases the risk of higher disease burden, such as intensified symptoms, hospitalizations, or other sequelae [5–7]. Diagnostic and therapeutic options remain limited, as the pathophysiological mechanisms underlying this syndrome have not yet been fully elucidated.

Various hypotheses have been proposed to explain the pathogenesis of post-COVID syndrome [8, 9]. Infection of the endothelium and hypercoagulability, along with their associated complications, have already been demonstrated in SARS-CoV-2 infections [10, 11]. Endotheliitis and the formation of microthrombi may lead to activation of coagulation factors and activation of a pro- and anti-inflammatory cytokine cascade, which in turn could cause perivascular inflammation and tissue damage [12, 13]. In this context, disturbance of oxygen transport into the muscles has also been discussed, which may reduce the anaerobic threshold of muscle activity [13]. Another hypothesis discusses the persistence of the virus, viral RNA, or viral proteins, which could lead to prolonged infections or autoimmunity [14–16].

Autoantibodies are considered potential triggers of tissue damage, particularly those functional antibodies against G protein-coupled receptors (GPCR) anchored in the cell membrane [17–19]. In particular, studies have described elevated levels of antibodies against β1- and β2-adrenergic receptors (β1/β2aR) as well as M3- and M4-muscarinic acetylcholine receptors (M3/M4mAcR) [20–22].

The β1- and β2-adrenergic receptors belong to the sympathetic nervous system and control central regulation of the “fight-or-flight” response. Activation of the β2 receptors in smooth muscle leads to relaxation, for example in the bronchi or blood vessels. Antibodies against these receptors could therefore affect autonomic regulation and in this way account for some of the functional disturbances observed in post-COVID syndrome [23, 24]. Muscarinic acetylcholine receptors are widely distributed in the central and peripheral nervous systems as well as in smooth muscle and exocrine glands, and mediate parasympathetic effects [25]. Thus, immunoadsorption has come into focus as a potential therapeutic intervention, with preliminary evidence from observational and pilot studies suggesting that reducing levels of autoantibodies, particularly against β-adrenergic and muscarinic receptors, may be a promising strategy in post-COVID syndrome. Improvements in fatigue, in physical performance, in autonomic symptoms, and in alleviation of cognitive deficits have been reported, although the results are heterogeneous and not all affected individuals benefitted from it [26–29]. However, a randomised, sham-controlled, patient-blind cross-sectional study involving 40 patients was recently published, which failed to demonstrate any clinically relevant improvements compared with the placebo group in patients with post-COVID syndrome [30]. Although immunoadsorption significantly reduced circulating autoantibodies and immunoglobulin G, no significant difference was observed in the primary endpoints, such as fatigue, functional capacity, cognitive performance or hand grip strength. These findings call into question the previous assumption that a reduction in autoantibodies leads directly to clinical improvement.

In light of these new, controlled data, current and previous observational studies require careful interpretation. This prospective exploratory study therefore investigates the efficacy of immunoadsorption in patients with post-COVID syndrome with respect to reductions in autoantibody levels and to changes in objective and subjective performance measures, with the findings interpreted in light of the current emerging body of evidence on immunoadsorption therapy.

## Patients, materials and methods

### Study design

Patients from the post-COVID outpatient clinic of the University Medical Center Rostock were included in this prospective exploratory clinical study. The study was conducted in accordance with international standards of good clinical practice (GCP) and the Declaration of Helsinki. All participants provided written informed consent after being fully informed. They received no compensation for their participation. The study was approved by the Ethics Committee of the University Medical Center Rostock (approval number A 2023-0104) and registered in the German Clinical Trials Register (DRKS00031554 and DRKS00033152).

The study endpoints were the biological and clinical efficacy as well as the safety of immunoadsorption. Biological efficacy was assessed based on reductions in immunoglobulin G and in antibodies against β1/β2aR and M3/M4mAcR. Clinical efficacy was assessed through the evaluation of core symptoms (fatigue, pain, cognitive impairment, cardiopulmonary complaints) using structured diaries, standardized functional tests (6 min walk test, handgrip strength measurement, spirometry, electrocardiography), and continuous monitoring of daily step count via smartwatch. Safety was assessed based on documented adverse events, including clinical symptoms, laboratory parameters, and vital signs.

### Study population

Men and women aged 18 to 60 years were eligible to participate in this study. Inclusion criteria were a diagnosis of post-COVID syndrome according to WHO criteria [2], detection of antibodies against the SARS-CoV-2 nucleocapsid protein (Elecsys Anti-SARS-CoV-2, Roche Diagnostics GmbH), a SARS-CoV-2 infection 12 or more weeks prior to study enrollment, and elevated antibody levels against β1/β2aR and/or M3/M4mAcR, defined as values above the reference limits established by the performing laboratory (IMD Berlin, Germany): β1aR > 15.0 U/ml, β2aR > 8.0 U/ml, M3mAChR > 6.0 U/ml, and M4mAChR > 10.7 U/ml.

### Exclusion criteria

Exclusion criteria included chronic infectious diseases such as hepatitis B or C, HIV, Lyme disease, tuberculosis, and acute EBV-infection, current immunosuppressive therapy, chronic cardiovascular diseases with NYHA class > 1, chronic neurological disorders such as multiple sclerosis, and currently treatment-requiring psychiatric conditions such as depression, participation in other clinical trials, and insufficient ability to participate. No prior organ transplantation or extracorporeal therapy was allowed.

### Immunoadsorption

Immunoadsorption was performed in inpatients in one week, on days 1, 2, 3, 5, and 7. Treatments were performed using reusable immunoadsorption columns on the LIFE 21^®^ apheresis device (Omni5; Miltenyi Biotec B.V. & Co. KG, Bergisch Gladbach, Germany). The columns nonspecifically adsorb immunoglobulin G and M, and to a lesser extent immunoglobulin E. Each procedure lasted between 2.5 and 4 h, processing each patient’s plasma volume twice [31]. Anticoagulation was performed using heparin and citrate, as is standard for this therapeutic system. Citrate anticoagulation was performed using ACD-A solution at a citrate-to-blood flow ratio of 1:22. The systemic concentration of ionized calcium was measured. If levels fell below 1.0 mmol/l and/or clinical symptoms consistent with hypocalcemia were present, the citrate-to-blood flow ratio was increased, and intravenous or oral calcium replacement was administered. Heparin was administered at the start of treatment (after ruling out heparin intolerance based on the patient’s medical history) as a bolus of 2000 IU; during treatment, a continuous infusion of 500–1000 IU/h (10–15 IU/kg/h, depending on body weight) was administered. The ACT (activated clotting time) was measured one hour after the start of treatment, and the infusion rates were adjusted accordingly (target ACT 180–200 s). If patients were considered at risk of bleeding, the initial heparin dose was reduced (heparin bolus 1,000 IU, continuous infusion 400 IU/h). Administration of immunoglobulins after treatment was not performed. In 17 of the 18 participants, due to insufficient peripheral venous access, a central venous catheter (CVC) was placed on the day of the first immunoadsorption session. In one patient, immunoadsorption was performed via peripheral vascular access.

### Randomization

**Fig. 1:**
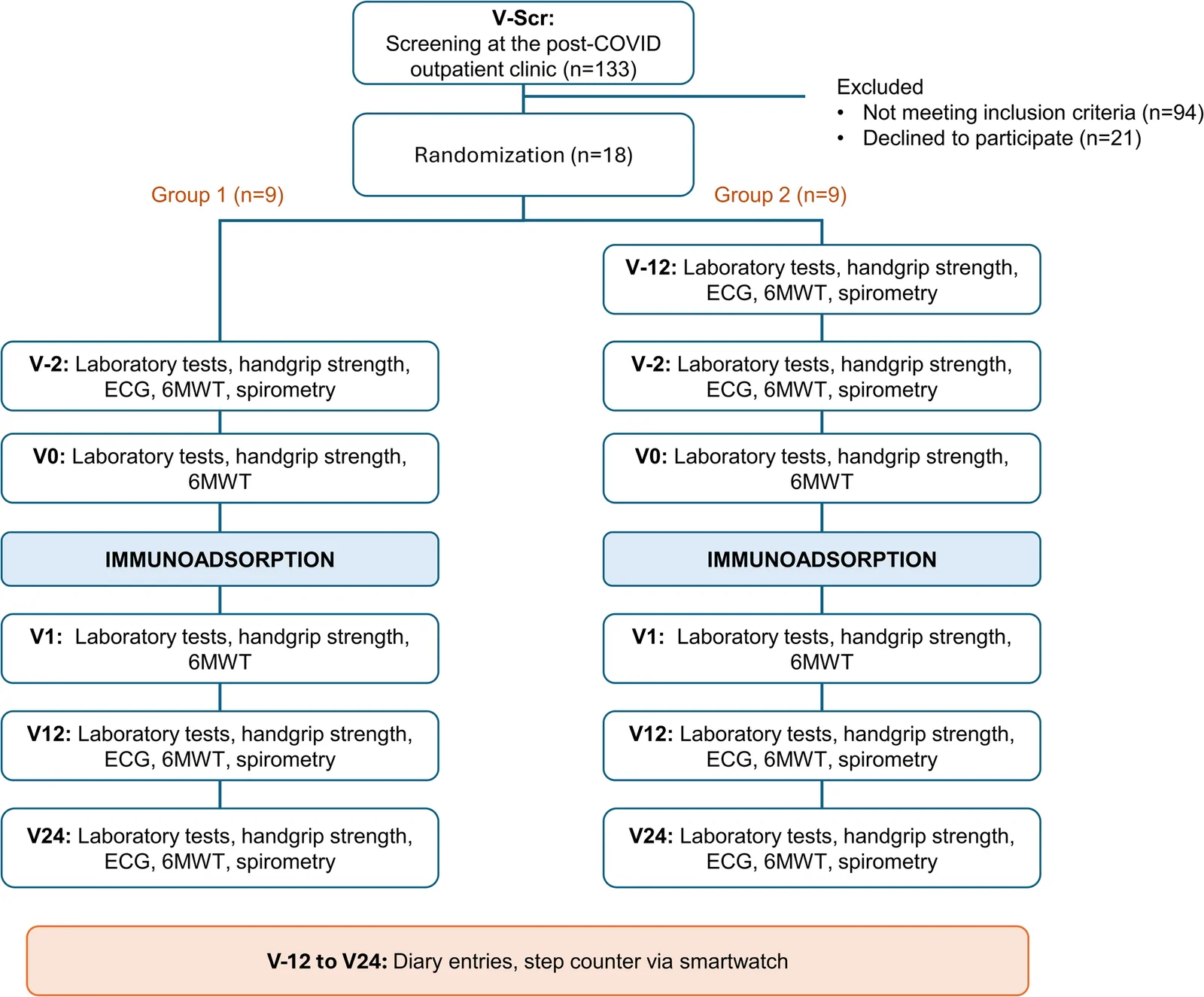
Clinical trial flowchart of the study

Since all participants were keen to start immunoadsorption as soon as possible, but the capacity for carrying out immunoadsorption was limited in terms of beds, equipment and staff, the 18 participants were randomized into two groups in a 1:1 ratio using sealed envelopes, with Group 2 having a longer pre-intervention observation period (Fig. 1).

### Laboratory analyses

Routine laboratory parameters, including coagulation markers, electrolytes, renal function parameters, albumin, and blood gas analysis, were assessed before, during, and after immunoadsorption in all participants. In all female participants, β-hCG levels were measured in blood prior to the first treatment session to exclude pregnancy.

Antibody concentrations against β1/β2aR and M3/M4mAcR were determined at time points V-Scr, V0, V1, V12, and V24 using an enzyme-linked immunosorbent assay (CE-IVD-certified ELISA, Cell-Trend, IMD-Labor Berlin, Germany). The lower limit of detection was < 2.5 U/ml for all measurements. The upper limit of detection was reduced by the laboratory from > 80 U/ml to > 40 U/ml at the time of the V24 assessment. Also, serum immunoglobulin G (IgG) levels were measured on each of the five treatment days, both before and after immunoadsorption. Serum and heparin plasma samples were frozen at −80 °C at V0 and V1.

### Visits and patient-reported outcomes

Participants were screened at time point V-Scr. In both groups, visits were conducted 2 weeks prior (V-2), immediately before (V0), immediately after the fifth cycle (V1), 12 weeks (V12) after immunoadsorption, and 24 weeks (V24) after immunoadsorption. In Group 2, additional visits were conducted 12 weeks prior (V-12) to immunoadsorption (Fig. 1).

Demographic data, as well as information on acute infections, vaccination status, pre-existing conditions, and risk factors were collected via a standardized questionnaire prior to study enrollment.

Participants were asked to record their cognitive and emotional activity, general activity, pain [32], fatigue, and overall symptom severity daily in structured diaries, each rated on a numerical scale from 0 to 10 throughout the study period. Subjective well-being was assessed using the Bell Disability Scale, a self-reported evaluation of physical capabilities [33]. Each participant was instructed to perform a 6-minute walk test (6MWT) at least every other day and was provided with a Fitbit Inspire 2 smartwatch (Fitbit Inc., USA) for objective documentation of step count. At each visit, participants underwent a standardized clinical examination, including handgrip strength measurement, electrocardiography (ECG), and a 6MWT. Spirometry was performed before and after each 6MWT (Fig. 1).

### Statistical analysis and graphical presentation

Statistical analysis was performed using the software R (R Core Team, https://www.r-project.org/) [34]. Graphical representations were generated using the *ggplot2* package (ggplot2, Springer International Publishing) [35].

Sample size was based on an expected reduction in antibodies against β1/β2aR and M3/M4mAcR in serum, as well as total IgG following immunoadsorption, taking into account data from the literature [26, 36]. Based on an anticipated effect size of approximately 80%, a required sample size of 10 patients per group was calculated for a significance level of *p* < 0.01.

Laboratory parameters (IgG and antibodies against β1/β2aR and M3/M4mAcR) were compared across visit time points using the nonparametric Wilcoxon signed-rank test. Laboratory values outside the quantifiable measurement range were replaced by the respective limit values for statistical analysis.

Clinical examination results and daily step counts before and after immunoadsorption were compared using the Wilcoxon signed-rank test. The subjective parameters (e.g., pain, fatigue, symptom burden, activity, Bell Disability Scale) were not suitable for significance testing due to the small sample size and the subjective nature of the assessments. Analysis was performed descriptively, and formal statistical tests were not conducted.

## Results

### Demographic data

Among the 133 patients who were screened between July 2022 and June 2024, 94 (70.7%) had elevated autoantibodies against β1/β2aR or M3/M4mAcR. Of these, 39 patients (29.3%) initially met the inclusion criteria. Four participants were unreachable by phone, two were enrolled in another clinical trial, five showed spontaneous symptom improvement, three had a new comorbidity or excluding medication, three perceived themselves as unable to participate, one became pregnant, and three withdrew consent without providing a reason. Ultimately, 15 women and 3 men were enrolled in the study. The mean age of the participants was 47 years (range 26–59, SD 9.8). Between the date of the infection that caused post-COVID syndrome and enrollment in the study an average of 25 (SD 8) months elapsed (Table 1).

**Table 1:** Demographic characteristics of the study participants

Characteristics	Group 1 (*n* = 9)	Group 2 (*n* = 9)
Median age in years (± SD)	48.7 (10.4)	45.9 (9.6)
Female sex, n (%)	6 (66.7)	9 (100)
Unable to work, n (%)	3 (33.3)	8 (88.9)
*SARS-Cov2 Vaccination status before disease onset*,* n* (%)		
No prior vaccination	1 (11.1)	1 (11.1)
3 prior vaccinations	8 (88.9)	7 (77.8)
4 prior vaccinations	0	1 (11.1)
*Triggering SARS-CoV-2 variant*,* n (%)*		
Alpha (08.02.2021–27.06.2021)	2	1 (11.1)
Omicron (20.12.2021–Present)	7 (77.8)	8 (88.9)
*Severity of SARS-CoV-2 infection*^*1*^, *n (%)*		
Mild	9 (100)	9 (100)
Prior rehabilitation, n (%)	7 (77.8)	7 (77.8)
*Risk factors*,* n (%)*		
Nicotine dependence	0	1 (11.1)
Overweight (BMI 25.0–29.9 kg/m²)	4 (44.4)	1 (11.1)
Class I obesity (BMI 30.0–34.9 kg/m²)	1 (11.1)	1 (11.1)

The data provided was collected using a standardised questionnaire

*SD* standard deviation, *BMI* body mass index

^1^ WHO Working Group on the Clinical Characterisation and Management of COVID-19 infection. A minimal common outcome measure set for COVID-19 clinical research. *Lancet Infect Dis.* 2020;20(8):e192–e197. doi: https://doi.org/10.1016/S1473-3099(20)30483-7

The most commonly reported symptoms among the 18 participants prior to study entry were fatigue (100%), non-restorative sleep (89%), cognitive impairments such as difficulty to concentrate (89%), word-finding troubles (83%), and memory disturbances (83%), exertional dyspnea (83%), palpitations (78%), headaches (78%), and muscle pain (78%).

### Laboratory results

**Fig. 2:**
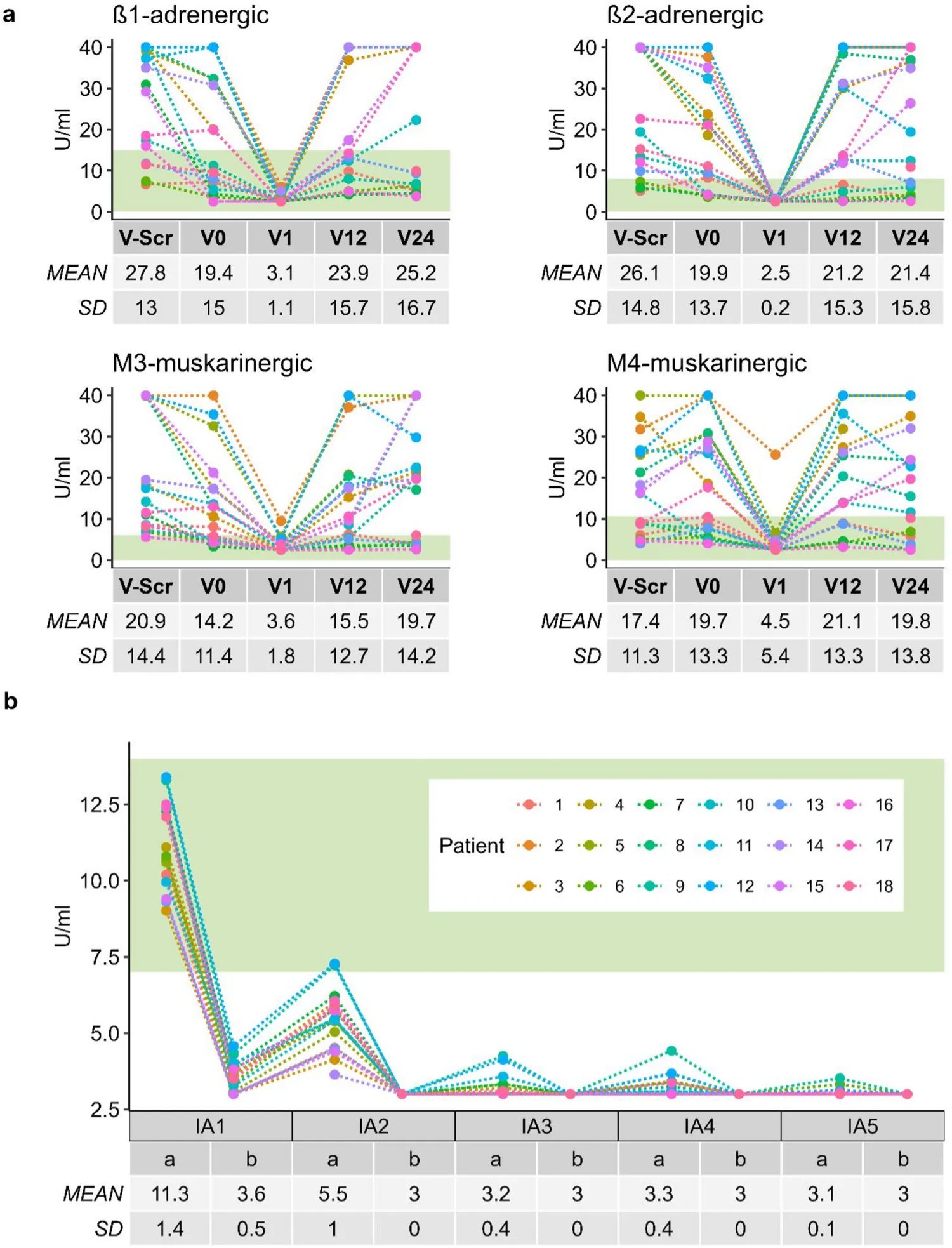
**a** Time course of ß1/ß2aR and M3/M4mAcR antibody concentrations; arithmetic means in U/ml; standard deviation in brackets. The normal range is highlighted in green (ß1aR < 15.0 U/ml, ß2aR < 8.0 U/ml, M3mAcR < 6.0 U/ml, M4mAcR< 10.7 U/ml). **b** Time course of immunoglobulin G concentrations over the five treatment days IA1–IA5; arithmetic means of IgG concentrations in g/l before (a) and after (b) immunoadsorption; standard deviation shown in brackets. The normal range is highlighted in green (7–16 g/l)

Antibody levels against β1/β2aR and M3/M4mAcR showed a significant decrease immediately after immunoadsorption (V1) compared to V-Scr and V0 (*p* < 0.01). Twelve and 24 weeks after immunoadsorption (V12, V24), both groups showed a significant increase in antibody levels, which returned to baseline (*p* < 0.01) (Fig. 2a).

Total IgG levels were within the normal range in all participants prior to treatment (mean 11.3 ± 1.4 g/l). After the second immunoadsorption session, IgG levels were near or below the analytical limit of detection (< 3 g/l) (Fig. 2b).

### Objective clinical parameters

**Fig. 3:**
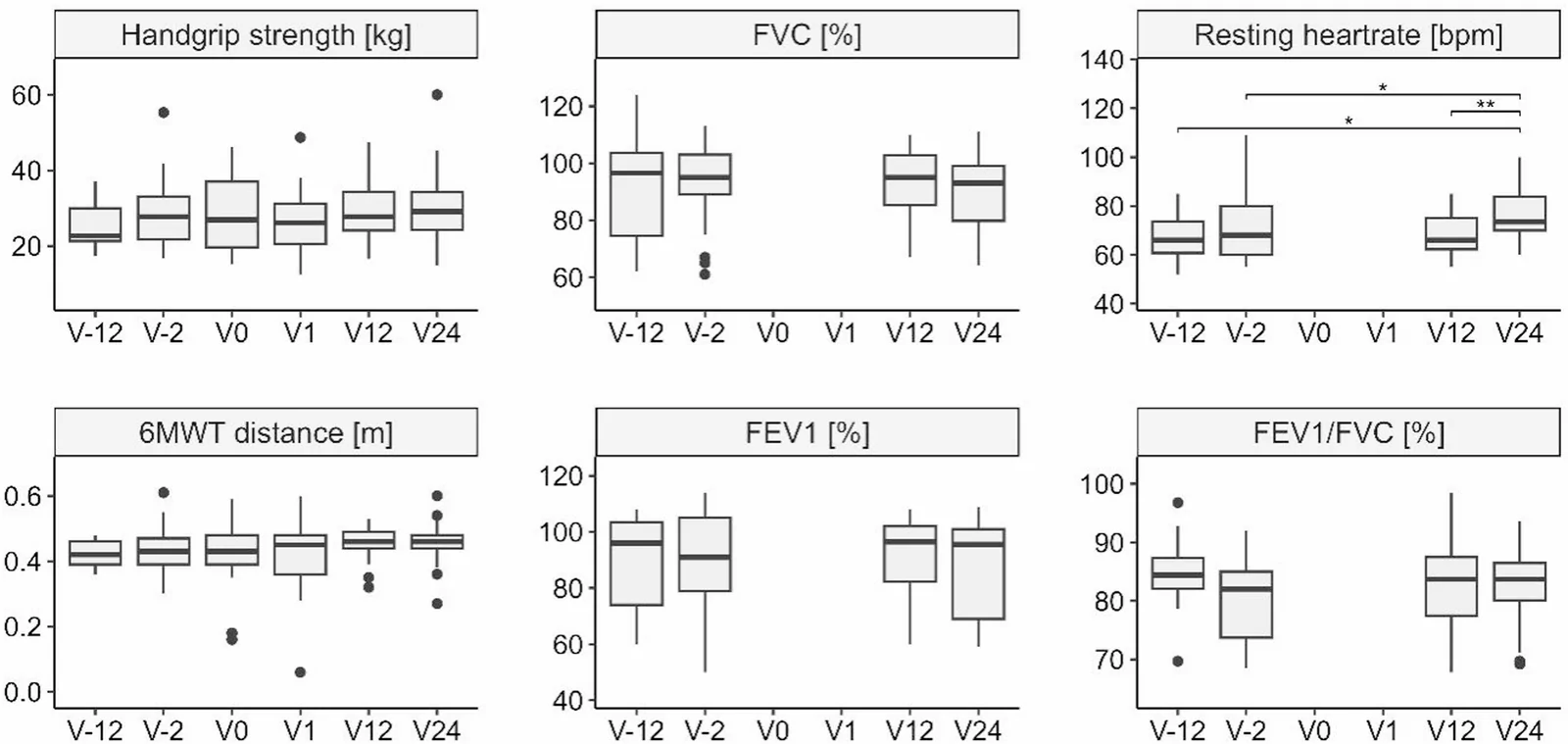
Boxplot of clinical test results (handgrip strength, 6-minute walk test, resting heart rate, and spirometry) across time points V-12, V-2, V0 (before immunoadsorption), V1, V12, and V24 (after immunoadsorption). Significant differences between individual time points are marked with *

Handgrip strength measurements, walking distances recorded in the outpatient clinic during the 6MWT, and spirometry tests (forced vital capacity [FVC], forced expiratory volume in 1 s [FEV1], and FEV1/FVC ratio) showed no significant differences between time points before (V-12, V-2, V0) and after immunoadsorption (V1, V12, V24) (*p* > 0.05). A separate analysis comparing clinical parameters measured at V1, corresponding to the time point at which autoantibody levels targeting β₁/β₂aR and M3/M4mAcR reached their nadir, with those measured at all other time points also showed no statistically significant differences (Fig. 3; Table 2). ECG findings showed no clinically relevant changes across the various follow-up visits; only the resting heart rate was significantly elevated at V24 compared with V-12, V-2, and V12 (*p* < 0.05).

**Table 2:** Results of clinical assessments at baseline and follow-up

	V-12	V-2	V0	V1	V12	V24
Handgrip strength (kg)	25.4 (6.6)	29.0 (9.7)	28.3 (9.5)	27.1 (9.3)	29.3 (8.4)	30.5 (10.6)
6MWT distance (m)	418 (43)	436 (36)	416 (109)	410 (117)	455 (60)	453 (75)
Resting heart rate (bpm)	68.3 (10.3)	68.7 (11.5)			67.3 (8.8)	74.8 (11.4)
*Spirometry*						
FVC (% predicted)	92.1 (17.1)	92.0 (14.4)			94.4 (12.0)	91.2 (12.4)
FEV1 (% predicted)	89.7 (15.1)	88.6 (18.4)			91.4 (14.1)	88.8 (15.8)
FEV1/FVC (%)	84.0 (7.0)	81.4 (7.8)			82.4 (7.2)	82.5 (6.0)

Data are presented as mean (SD)

*6MWT* 6-minute walk test, *bpm* beats per minute, *FEV1* forced expiratory volume in 1 s, *FVC* forced vital capacity, *IA* Immunoadsorption, *SD* standard deviation

**Fig. 4:**
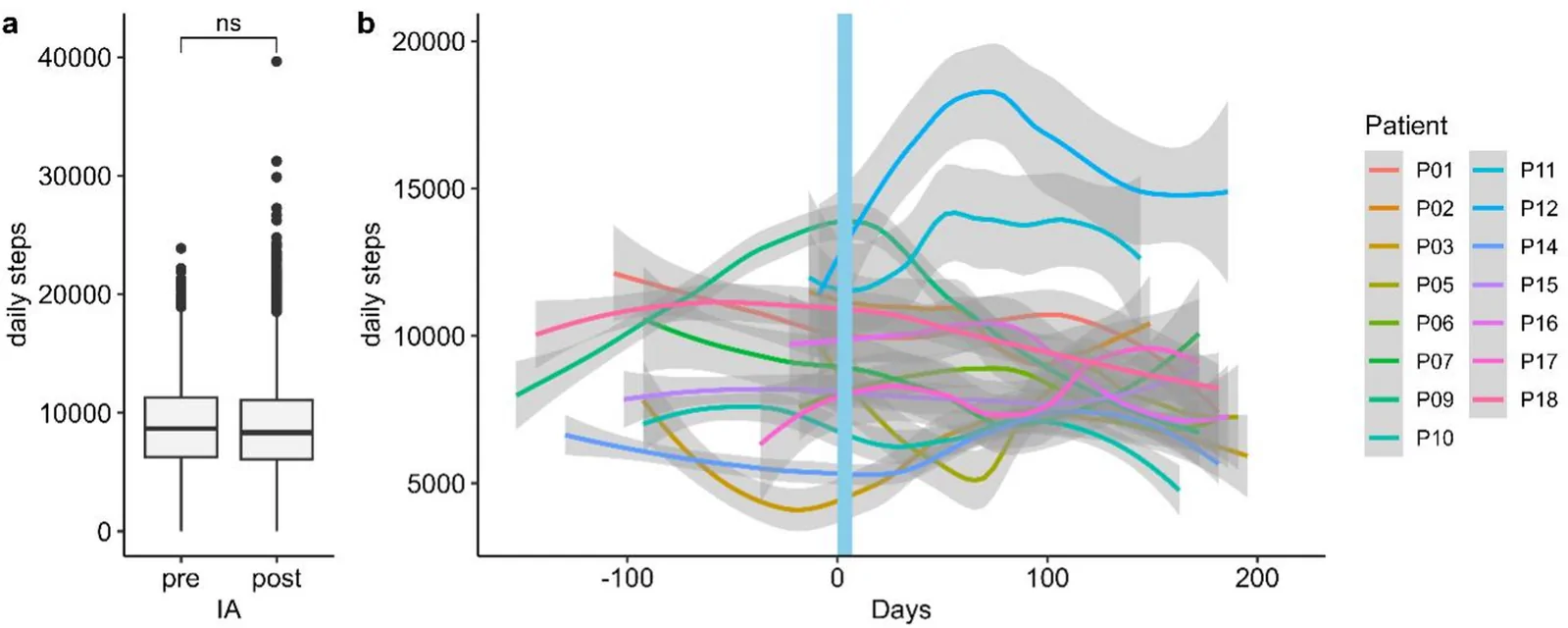
**a** Boxplot of daily step counts comparing values before immunoadsorption (V-12 to V0) and after immunoadsorption (V1 to V24). **b** Time course of daily step counts in 18 participants, shown with smoothed curves using locally estimated scatterplot smoothing (LOESS) regression

The analysis of step count data recorded by the smartwatch was based on data from 15 participants. The mean daily step count was 9029 (± 3896) steps/day before immunoadsorption (V-12 to V0) and 8944 (± 4507) steps/day after immunoadsorption (V1 to V24). The difference was not statistically significant (*p* > 0.05; Fig. 4a and b; Table 3). In the correlation analysis, no significant association was found between changes in circulating autoantibody levels and daily step counts (Pearson correlation, *R* = 0.099, *p* = 0.483).

**Table 3:** Results from the diary data

	M-3	M-2	M-1	M1	M2	M3	M4	M5	M6
Daily steps	8572 (3688)	8555 (4179)	9616 (4296)	8919 (4416)	9543 (4723)	9400 (4683)	8803 (4223)	9023 (4749)	7921 (3963)
6MWT (m)	424 (105)	427 (121)	424 (96)	442 (101)	470 (88)	466 (97)	477 (99)	490 (90)	463 (93)
Mental activity	4.7 (1.90)	4.7 (1.91)	4.7 (2.27)	5.0 (2.37)	5.3 (2.45)	5.3 (2.33)	5.5 (2.47)	5.4 (235)	5.7 (234)
Emotional activity	4.8 (2.16)	4.5 (2.23)	5.1 (2.27)	5.3 (2.15)	5.4 (2.32)	5.4 (2.32)	5.6 (2.29)	5.6 (221)	5.7 (231)
Total activity	5.6 (2.42)	5.5 (2.36)	5.8 (2.42)	5.8 (2.25)	5.9 (2.36)	5.9 (2.25)	6.1 (2.40)	6.1 (2.19)	6.2 (2.38)
Pain	5.8 (1.83)	5.5 (1.80)	5.0 (2.26)	3.6 (2.35)	3.8 (2.48)	4.0 (2.57)	4.3 (2.59)	4.4 (2.48)	4.8 (2.39)
Fatigue	5.4 (2.15)	5.4 (2.20)	6.1 (1.85)	5.2 (1.99)	5.0 (1.91)	5.1 (1.91)	4.7 (2.21)	4.9 (2.16)	5.4 (234)
Overall symptoms	5.6 (2.16)	5.4 (2.21)	6.0 (1.87)	5.3 (2.03)	5.0 (2.13)	5.2 (1.93)	4.8 (2.29)	4.9 (2.23)	5.4 (2.43)
Bell score	40 (15)	41 (16)	42 (16)	51 (20)	54 (19)	53 (19)	50 (20)	50 (22)	53 (20)

Monthly observation periods relative to the intervention (M-3 to M-1 = months before the intervention; M1 to M6 = months after the intervention). Data are presented as mean (SD)

### Diary data

Diary data from 16 participants were available for analysis. The mean values of the parameters recorded in the diary were summarized over a four-week period and are presented in Table 3. The mean distance walked in the home-based 6MWT was 424.5 (SD 97.8) before immunoadsorption and increased to 468 (SD 95.4) after intervention. Participants perceived a slight improvement in several symptoms compared to baseline: pain scores decreased from 5.5 (SD 2.0) to 4.1 (SD 2.5), fatigue decreased from 5.6 (SD 2.0) to 5.0 (SD 2.1), overall symptom burden decreased from 5.8 (SD 2.0) to 5.1 (SD 2.2), and physical functioning as assessed by the Bell Disability Scale improved from 39.8 (SD 15.3) to 52.4 (SD 20.0) points. Additionally, a slight increase in cognitive, emotional, and overall activity was reported compared to baseline.

In the correlation analysis, no significant association was found between changes in circulating autoantibody levels and subjectively reported symptoms (Pearson correlation, *R* = −0.11 to 0.13; *p* > 0.05).

### Safety

Three adverse events occurred during immunoadsorption. One participant experienced an arterial mispuncture during CVC placement, which resolved without further complications. Another participant developed a panic attack on the final day of the inpatient stay, though not during the immunoadsorption therapy. A third participant developed a superficial thrombophlebitis at the puncture site in the right antecubital fossa two days after the final immunoadsorption session, which was successfully treated with oral antibiotics.

No allergic reactions, cardiovascular or circulatory disturbances, bleeding complications, CVC-related infections, or clinically relevant changes in laboratory parameters were observed during immunoadsorption in any participant.

During the observation period (V1-V24), three participants experienced mild SARS-CoV-2 infections, two of whom were treated with Paxlovid^®^ (nirmatrelvir/ritonavir). Four other participants reported febrile respiratory tract infections, which were successfully managed on an outpatient basis.

## Discussion

To date, there is only limited evidence for the clinical efficacy of immunoadsorption in the treatment of post-COVID syndrome. It is not among the treatments recommended by current German guidelines [37]. Nevertheless, the treatment is offered in some settings as a non-reimbursable individual health service (Individuelle Gesundheitsleistungen, IGeL), with total costs estimated to exceed 16,000 euros for a full treatment course [38]. However, due to the disease burden, which is substantial for many patients, there is often a high demand for therapeutic interventions that extend beyond symptomatic treatment. In light of the growing critical assessment of previous observational data and the new controlled evidence from the literature—which has so far failed to confirm clinically relevant efficacy—this prospective exploratory study investigates the effectiveness of immunoadsorption in post-COVID syndrome with regard to the reduction of autoantibodies as well as objective and subjective performance, contributing to the current evidence base.

Despite the approximately 400 patients seen annually in our post-COVID outpatient clinic, recruitment efforts had limited success. The main reason was that only a subset of patients received a confirmed diagnosis of post-COVID syndrome [2]. Furthermore, many patients did not meet inclusion criteria, primarily due to relevant comorbidities. Among the 133 screened patients, antibodies against β1/β2aR and M3/M4mAcR were not detectable in 29%. Additionally, several patients resigned at short notice for personal reasons, due to safety concerns, or because of spontaneous clinical improvement. As a result, only 18 instead of the planned 20 participants could be included in the study.

Our study population showed a representative distribution of demographic characteristics (Table 1). Consistent with the literature, the majority of our patients were women of middle age, with a symptom profile typical of post-COVID syndrome [5, 39]. The most commonly reported symptoms among our participants were four core symptoms: fatigue, cardiopulmonary complaints, cognitive impairments, and pain.

Adverse events (AEs) such as hypotension, access-related problems, paresthesia, and urticaria are reported in 8.4% of registered immunoadsorption procedures [40]. The risks observed in our study, such as arterial mispuncture during CVC placement and superficial thrombophlebitis after peripheral access, did not raise additional safety concerns.

Immunoadsorption rapidly and significantly reduced IgG and circulating antibodies against β1/β2aR and M3/M4mAcR in our patients. The subsequent increase in antibody concentrations to baseline levels during follow-up visits at 3 and 6 months (V12, V24) suggests that there was no sustained reduction in antibody titers, and therefore possibly no long-term clinical benefit is to be expected. The absence of correlations between changes in autoantibody titers and both objectively measured and subjectively reported outcomes supports the hypothesis that autoantibodies may not be the primary driver of symptoms in our patient cohort.

If autoantibodies are indeed disease drivers, a long-term therapeutic effect cannot be anticipated from immunoadsorption. While immunoadsorption effectively removes circulating antibodies, complement, and pro-inflammatory cytokines, it does not eliminate the antibody-producing B cells [41]. This aligns with clinical experience in other autoimmune diseases, such as multiple sclerosis, systemic lupus erythematosus, and rheumatoid arthritis, where autoantibodies are rapidly cleared, leading to transient clinical improvement, until antibody levels rise again after some weeks [42]. In rheumatoid arthritis and multiple sclerosis, a clinical response has been observed in 48% and 88% of cases respectively within a follow-up period of up to 12 weeks after immunoadsorption [43]. In contrast, our data do not show such a pronounced clinical efficacy in post-COVID patients, despite the significant reduction in autoantibodies and immunoglobulins. This suggests that the observed symptom burden may be driven by mechanisms beyond circulating autoantibodies.

In our participants, no significant improvement in objectively measured physical performance was observed after immunoadsorption, including 6MWT distance, daily step count, handgrip strength, resting heart rate on ECG, and spirometry.

Since only a limited number of validated objective tests are currently available for assessing disease activity in post-COVID conditions, subjective assessments in terms of structured diaries were also incorporated into data collection. Diary analysis revealed a minor reduction in pain and overall symptom burden, along with a decrease in fatigue and increased perception of physical activity. These findings suggest a partial subjective improvement, which, however, did not correlate with objective performance gains. Furthermore, no significant association was found between changes in circulating autoantibody levels and subjectively reported symptoms in the correlation analysis. This suggests that the observed subjective changes may not be directly mediated by the reduction of autoantibodies. Interestingly, the home-based 6MWT showed increases in walking distance after immunoadsorption, whereas no corresponding improvement was observed in the setting of the standardized clinical consultation for any participant. This discrepancy between home- and clinic-based results highlights the methodological challenges in interpreting subjective self-reports compared to objective performance measurements.

To date, one randomized trial, several small-scale studies and case series have been published on immunoadsorption in post-COVID patients with elevated antibodies against β-adrenergic and/or muscarinic receptors. Similar to our study, these publications report that immunoadsorption can temporarily reduce autoantibodies and immunoglobulin G [26–29]. In all these investigations, including our own, five treatment sessions were performed within a period of less than 14 days. However, the studies show heterogeneous findings regarding clinical efficacy. Due to different questionnaires and inconsistent objective measured parameters, the results are not directly comparable. The questionnaires and scores used in these studies are summarized in Table 4.

**Table 4:** Comparison of assessment instruments used in the referenced studies and this study

	Stein et al. [26]	Giszas et al. [27]	Ruhe et al. [29]	Anft et al. [28]	Stortz et al. [30]	This study
Global assessment scale (GAS)	–	✓	–	–	–	–
Patient health questionnaire-9 (PHQ-9)	–	✓	✓	–	–	–
Fatigue assessment scale (FAS)	–	✓	✓	–	–	–
Montreal cognitive assessment (MoCA)	–	✓	–	–	✓	–
36 item short-form survey (SF-36)	✓	–	–	✓	–	–
Fatigue severity scale (FSS)	✓	–	–	–	–	–
Bell score	✓	–	–	✓	✓	✓
DePaul symptom questionnaire (DSQ-PEM)	✓	–	–	–	–	–
Composite autonomic symptom score (COMPASS-31)	✓	–	–	✓	–	–
Hand grip strength (HGS)	✓	–	✓	✓	✓	✓
Reactive hyperemia index (RHI)	✓	–	–	–	–	–
Post-COVID-syndrome score	–	–	✓	–	–	–
Sit-to-stand repetitions	–	–	✓	–	–	–
European quality of life 5 dimensions (EQ-5D)	–	–	✓	–	–	–
CERAD+ test battery^a^	–	–	–	✓	–	–
Chalder fatigue scale	–	–	–	✓	✓	–
Canadian consensus criteria for ME/CFS (CCC)	✓	–	–	✓	–	–
Munich Berlin symptom questionnaire (MBSQ)	–	–	–	✓	–	–
Epworth sleepiness scale (ESS)	–	–	–	✓	–	–
Post-COVID functional scale (PCFS)	–	–	–	–	✓	–
Multidimensional fatigue inventory (MFI-20)	–	–	–	–	✓	–
6-minute walk test (6MWT)	–	–	–	–	–	✓
Spirometry	–	–	–	–	–	✓
Electrocardiography (ECG)	–	–	–	–	–	✓
Daily symptom diary	–	–	–	–	–	✓
Daily step count	–	–	–	–	–	✓

✓ = instrument used; – = instrument not used

^a^CERAD+ Test Battery (Consortium to Establish a Registry for Alzheimer’s Disease)

The first randomized, patient-blinded, sham-controlled crossover trial investigating immunoadsorption in 40 post-COVID syndrome was recently published. Although immunoadsorption significantly reduced circulating autoantibodies against G-protein-coupled receptors as well as IgG levels, no clinically relevant improvements were observed compared with sham treatment regarding fatigue, functional status, cognitive performance, or handgrip strength. The authors therefore concluded that, despite successful autoantibody depletion, immunoadsorption did not provide a clinically meaningful benefit in post-COVID syndrome [30].

In another one study, 14 of 20 patients reported a clinically relevant improvement of at least 10 points in the SF-36 Physical Functioning Score after immunoadsorption [26]. Additionally, sustained improvements were observed in fatigue, post-exertional malaise and pain, as well as in cognitive, autonomic, and immunological clinical parameters. In another study [27], a transient improvement of symptoms was documented in one of two patients treated with immunoadsorption. One patient reported a clear improvement in physical and mental condition after the fifth treatment cycle. However, two months after treatment, symptom severity had returned to baseline levels. The other patient reported only a minor subjective improvement. In a further observational study [28], all 12 post-COVID patients showed neuropsychological improvements and slightly increased handgrip strength 30 days after immunoadsorption. However, subjective symptoms remained unchanged. In a case series of 10 patients with post-COVID syndrome, no clinically relevant changes in mental or physical health were observed following immunoadsorption [29].

Despite a significant reduction in autoantibodies observed in several studies – including the present study and the recently published, sham-controlled study [43] – no clinically relevant benefit of immunoadsorption has been demonstrated in the majority of studies.

In addition to immunoadsorption, therapeutic plasma exchange (TPE) has also been investigated in patients with post-COVID syndrome. In a randomised, double-blind, placebo-controlled Phase II trial involving 50 patients, TPE proved to be a safe procedure; however, compared with the placebo group, it did not lead to any significant improvements in functional capacity, symptoms, cognitive function or health-related quality of life [44]. These findings underscore the need to critically evaluate the clinical efficacy of using apheresis procedures to treat post-COVID syndrome, particularly as the reduction of autoantibodies alone is not sufficient to achieve sustained clinical improvement.

In our study, we used a smartwatch step counter to obtain an objective assessment of daily activity. A systematic review of 32 observational studies on daily activity showed that healthy young adults typically take approximately 7000**–**13,000 steps per day [45]. We hypothesized that a reduction in fatigue and pain would lead to an increase in daily activity resulting in increased step counts. Reduced pain and fatigue were reported by 50% of our participants, but this was not accompanied by an increase in daily steps. Notably, after immunoadsorption, nine participants reported pain scores above 5 on the numerical rating scale, indicating severe pain, yet still walked more than 10,000 steps per day.

Furthermore, our participants reported Bell Disability Scale scores of 39.8 (SD 15.3) points before immunoadsorption, corresponding to moderate functional impairment with only a few hours of light activity per day. Despite this, they achieved an average of 9029 steps (SD 3896) per day. After immunoadsorption, participants reported higher Bell scores (52.4, SD 20.0), indicating improved self-perceived function, although their daily step count did not increase.

This discrepancy between objectively measured activity, comparable to that of healthy adults, and subjectively reported severe symptoms such as pain and fatigue should be pursued in future studies.

In addition to subjective parameters, our study also collected objectively measurable outcomes, which should be considered in future studies. Precise methods such as cardiopulmonary exercise testing and lactate measurements during bicycle ergometry enable changes in physical capacity to be reliably assessed. Furthermore, modern wearables, such as wrist-worn devices, offer the opportunity to measure a wide range of physiological parameters, including step count and heart rate, as well as oxygen saturation, skin temperature, ECG, blood pressure and sleep patterns. These multimodal data not only provide insights into improvements in physical fitness but also offer a more comprehensive understanding of patients’ quality of life and daily routine.

Our study has several methodological limitations. As previously mentioned, only 18 participants were enrolled instead of the originally planned 20, which was due to some patients withdrawing prior to the start of this invasive procedure. A placebo-controlled design would have required CVC placement in patients with poor peripheral venous access, to simulate blood exchange without immunoadsorption. Moreover, due to the complexity in assessing therapeutic response and the subjective nature of widespread symptoms, a very large sample size would have been required to detect notable improvement in symptoms, making funding somewhat difficult. After careful considerations a placebo group could not be not established in our study for ethical and logistical reasons.

Although immunoadsorption has proven to be safe and effective in removing immunoglobulin G and specific autoantibodies in our and in other studies [26–29], we did not observe any objectively measurable, significant improvement in physical performance in patients with post-COVID syndrome. Nevertheless, some participants reported at least minor subjective improvements in their diaries, particularly regarding pain and fatigue.

From a clinical perspective, these results indicate that careful patient counseling is warranted regarding this experimental intervention, which is currently offered outside established, guideline-based medical care. Given the often high expectations of affected patients, transparent communication regarding the limited evidence base, potential risks, and frequently self-funded costs of this procedure is essential.

## Data Availability

The datasets generated and/or analysed during the current study are not publicly available due to ethical and privacy restrictions.
